# Presentation of intestinal malrotation and midgut volvulus in adults: Case report & literature review

**DOI:** 10.1016/j.ijscr.2020.06.066

**Published:** 2020-06-20

**Authors:** Hassan Dehaini, Rakan Nasser Eldine, Samer Doughan, Mohammad Khalifeh, Hala Khasawneh, Hero Hussain, Eman Sbaity

**Affiliations:** a Division of General Surgery, Department of Surgery, Faculty of Medicine, American University of Beirut, Beirut, Lebanon; b Division of Vascular & Endovascular Surgery, Department of Surgery, Faculty of Medicine, American University of Beirut, Beirut, Lebanon; c Division of Diagnostic Radiology, Department of Radiology, Faculty of Medicine, American University of Beirut, Beirut, Lebanon

**Keywords:** Late presentation, Congenital disease, Intestinal obstruction, Ladd procedure, Case report

## Abstract

**Introduction:**

Malrotation is considered a newborn disease. This case report sheds light on the rare, but possible late presentation of malrotation in adulthood, which if missed, can leave the patient in a detrimental state.

**Presentation of case:**

28-year-old female presented in critical state with acute abdomen. Computed tomography abdomen/pelvis showed midgut volvulus, requiring urgent laparotomy. The patient’s bowels were discolored, yet they normalized upon detorsion, except for a small portion, which was equivocal and left for observation. Ladd’s bands were excised, and the abdomen was closed with Bogota bag for re-exploration. The patient underwent two more laparotomies to observe the intestinal segment until it was back to normal. Ladd procedure was then completed, and an absorbable mesh was applied. Follow-up of 20 months has been uneventful, except for a small, asymptomatic, incisional hernia.

**Discussion:**

Malrotation in adults is often missed due to its subacute, nonspecific presentation. It is often diagnosed by CT abdomen, which shows inversion or vertical positioning of the superior mesenteric vessels. Symptomatic, but stable patients, can undergo laparoscopic Ladd procedure, which carries the benefit of less length of stay. While an incidental malrotation can be prophylactically operated on, correcting asymptomatic malrotation beyond age of 20 is ineffective and possibly harmful.

**Conclusion:**

Intestinal malrotation presenting in an adult should be on the differential diagnosis when dealing with abdominal pain, especially in the context of small bowel obstruction in a virgin abdomen. It is vital to consider a patient’s age prior to prophylactically operate on malrotation discovered incidentally.

## Highlights

• Intestinal malrotation is uncommon in adulthood and is often missed.
• Computed tomography scan of the abdomen is the diagnostic tool of choice.
• Prompt intervention by surgical exploration is crucial for patient survival.
• Laparoscopy is challenging in diagnosed volvulus or acute bowel obstruction.
• Correcting asymptomatic malrotation after 20-years-old is not recommended.

## 1 Introduction

Intestinal malrotation is the failure of the midgut to rotate 270° counterclockwise during development. Consequently, the duodenum and cecum are joined together through Ladd’s bands, precipitating midgut volvulus, intestinal obstruction and ischemia [2]. The incidence of malrotation is around 1 in 500 live births [1], while symptomatic malrotation occurs in 1 in 6000 births instead [3].

Although malrotation is considered a disease of the newborn and pediatric population, cases occurring in adults have occasionally been reported, with recent analyses revealing that up to 48% of malrotation cases can present in adulthood [4]. Our case report is about a 28-year-old female whose malrotation went unnoticed, despite recurrent abdominal pain, until her emergent presentation to our academic institution. This work is being reported in line with the SCARE 2018 criteria [5].

## 2 Case presentation

A 28-year-old female, non-smoker and non-alcohol drinker, was transferred from a hospital to our institution’s emergency department by an ambulance for severe, acute abdominal pain. The pain was diffuse and progressively worsening over the past few days. It was associated with nausea and one episode of non-bilious, non-bloody emesis. She denied changes in her bowel movements, fever and chills.

The patient’s past medical, surgical and family history were non-contributory, except for a history of episodic, recurrent, vague abdominal pain that occurred every few months over the past few years prior to presentation and was responsive to analgesics. The patient was not taking medications nor using illicit drugs.

On physical examination, the patient was in acute distress, screaming in pain. She had a heart rate (HR) of 126 beats per minute (bpm) and a blood pressure of 107/85. Her abdomen was diffusely tender to palpation, tense and distended.

**Fig. 1:**
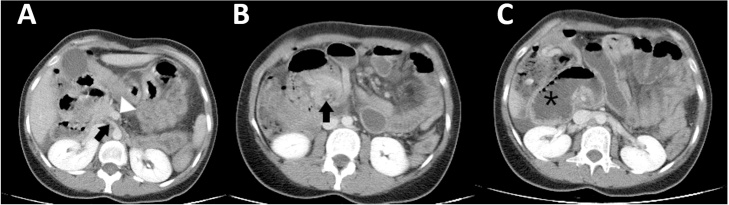
Axial contrast-enhanced CT images of the upper abdomen. **(A)** Superior mesenteric artery (black arrow) is abnormally located to the right of the superior mesenteric vein (white arrowhead). **(B)** Abnormally positioned small bowel around the SMA (black arrow) with a characteristic swirling pattern, consistent with midgut volvulus. **(C)** Small bowel dilatation (asterisk) secondary to small bowel obstruction due to midgut volvulus.

The patient’s blood work was pertinent for metabolic and lactic acidosis: pH 7.29, bicarbonate 15 mEq L^−1^, and lactic acid 3.69 mmol L^−1^. A computed tomography (CT) scan of her abdomen and pelvis was done at a peripheral hospital 3 h prior to presentation and was reviewed by our team. It showed right-to-left inversion of superior mesenteric artery (SMA) and vein, as well as swirling intestines, consistent with malrotation and midgut volvulus (Fig. 1). Due to the patient’s unstable condition, we forfeited further investigations, based our judgement on the CT imaging done at the previous hospital and brought in by the patient, and rushed her for operative exploration after obtaining the patient’s consent.

**Fig. 2:**
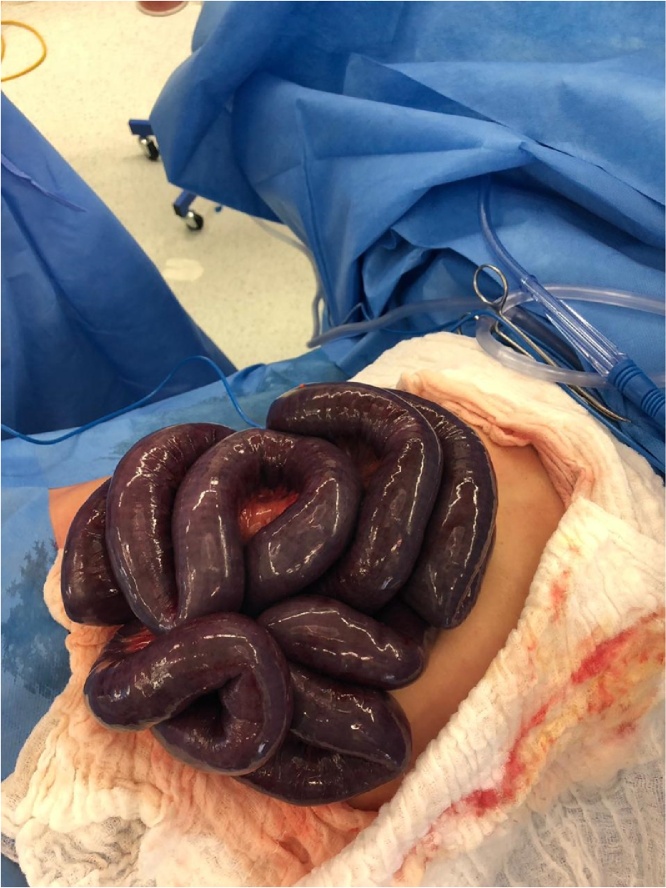
Laparotomy showing ischemic bowels.

**Fig. 3:**
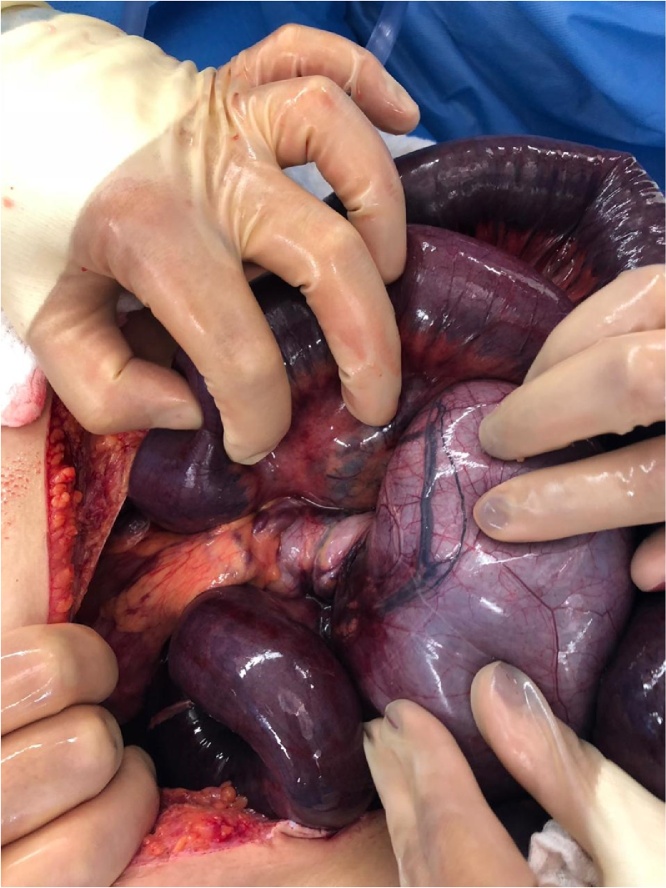
Rotation of the mesentery over Ladd bands resulting in volvulus.

Under general anesthesia, exploratory laparotomy was done by an attending general surgeon and a chief resident with 5 years of specialized training. It revealed bluish discoloration of the small bowels with areas that were significantly dark (Fig. 2). The mesentery was twisted, resulting in secondary ischemia (Fig. 3). The bowels and mesentery were untwined, which caused the intestines to resume their healthy color and peristalsis. However, a length of about 40 cm remained borderline-ischemic, marked with punctate hemorrhage, yet peristaltic with positive Doppler signal. Thus, we preferred to have a second look in 24 h.

Meanwhile, the veins along the ascending and transverse mesocolon were noted to be tortuous. We avoided ligating these tortuous veins in case they were serving as an alternate drainage for the small bowel, which instead would cause congestion and worsening of small bowel ischemia. Ladd's bands were then excised, and the abdomen was closed with Bogota bag after irrigating it with warm water.

Postoperatively, the patient was transferred to the surgical intensive care unit (SICU), where she received vasopressors and antibiotics. Her laboratory workup showed leukocytosis (WBC 11600, 85%) and persistent metabolic and lactic acidosis (HCO3^−^ 14, lactic acid 4.7 mmol L^−1^). Six hours postoperatively, the patient developed decreased urinary output, worsening abdominal exam and exacerbated lactic acidosis (lactic acid 7.13 mmol L^−1^). Tissue reperfusion injury or necrosis of the borderline segment of the small intestines were suspected, therefore, the patient underwent emergent re-exploration.

**Fig. 4:**
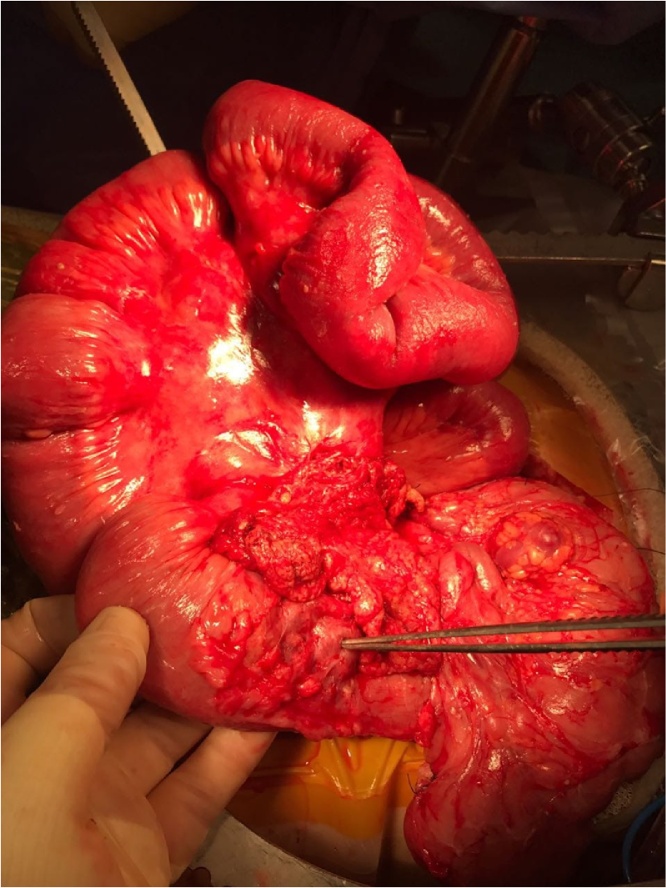
Revascularization of the bowel after de-rotating the mesentery by surgical team. Note the dilated veins at the walls of the colon.

Intraoperatively, the small intestine that showed borderline viability in the first surgery, looked viable, except for a 20 cm stretch whose viability was still questionable. The majority of the bowel resumed Its viable color, peristalsis and had palpable mesenteric pulses (Fig. 4). Another laparotomy was planned in 48 h.

Over the next 48 h, the patient was hemodynamically stable, and her laboratory workup normalized. In the last laparotomy, all the bowels were viable without signs of persistent ischemia. Ladd procedure was completed; small bowel mesentery was further broadened; its serosa was fixed to the retroperitoneum and the appendix was removed. The abdominal wall edges could not be fully approximated; thus, the defect was closed using 30 × 30 cm Vicryl mesh. Finally, an abdominal binder was applied postoperatively. The remaining hospital stay was noneventful; the patient was discharged home 5 days later.

The patient was initially followed-up clinically every few weeks, then every 6 months for a total of 18 months. Patient has been doing well, tolerating diet and gaining weight. She has developed, however, a small, reducible midline incisional hernia with minimal discomfort, despite adhering to the abdominal binder and avoiding heavy lifting for 6 months as we recommended. Nevertheless, the patient has been satisfied with the outcome.

## 3 Discussion

Cases of intestinal malrotation in adulthood have been reported in the scientific literature. Nevertheless, this entity has often eluded practitioners since it has long been associated with the pediatric population, as in the case of our patient, who sought medical advice repeatedly for mild to moderate, recurrent abdominal pain, to no avail. Our case report emphasizes the importance of considering late presentation of intestinal malrotation as part of the differential diagnosis for unexplained abdominal pain to avoid the highly morbid intestinal ischemia. Furthermore, it shows that conserving the intestines in case of uncertainty is a valid option in place of intestinal resection, especially in a young patient.

Intestinal malrotation is a congenital abnormality that arises from disrupting the 270° counterclockwise midgut rotation during the embryonic period [6]. Intestinal malrotation exists in multiple forms; the most common are nonrotation, reversed rotation and incomplete rotation [7]. Incomplete rotation is the classical malrotation that is usually described, and is due to rotating 180° counterclockwise instead of 270°, leaving the cecum in the right upper quadrant [7]. Intestinal malrotation can be complicated with volvulus around a narrowed mesentery [8], or bowel obstruction by misplaced peritoneal folds or the SMA [7].

In pediatric population, the cardinal sign of intestinal malrotation is bilious emesis of sudden onset [4]. This is often recognized, facilitating diagnosis within hours to days. The diagnosis is approached through three modalities: the initial step is a plain abdominal radiograph, which commonly yields normal results [9], but can rarely show signs of duodenal obstruction or intestinal malposition [10]. Abdominal ultrasonography is another diagnostic tool that is used, in which a retro-mesenteric position of the duodenum’s third segment indicates intestinal malrotation [11]. Nevertheless, upper gastrointestinal imaging (UGI), whose sensitivity is 96%, remains the “gold standard”, where the “corkscrew appearance” of the duodenal-jejunal junction (DJJ) and its deviation to the left of the vertebral body suggest malrotation [12].

In adults, malrotation causes chronic, nonspecific abdominal pain that is usually missed for months to years [4,13], as in the case of our patient, since it is low on the differential diagnosis. A CT abdomen with oral and intravenous (IV) contrast showing inversion or vertical positioning of SMA and superior mesenteric vein (SMV) is diagnostic of intestinal malrotation [14].

Ladd procedure remains the standard procedure for complicated malrotation. It constitutes of four primary steps: counterclockwise detorsion of the intestines, followed by dissection of Ladd’s bands to relieve the obstruction, broadening the small intestines mesentery to prevent recurrence, and an incidental appendectomy [7]. It can be done through either laparotomy or laparoscopy. Surgeons should be attentive to the dilated veins draining the colon and not ligate them, because they are the main drainage of the colon in cases of malrotation.

Several case series have shown that laparoscopic Ladd procedure causes decreased length of stay (LOS) and postoperative nasogastric decompression, with a rate of conversion to laparotomy between 2% and 33% [[15], [16], [17]]. It is noticeable, however, that the physical status profile of those who underwent laparoscopy was markedly better than those who underwent laparotomy, hence laparoscopy is yet to be recommended for cases with acute surgical abdomen or known volvulus [18].

As for asymptomatic malrotation, its correction prophylactically is yet to be settled in stone. The probability of asymptomatic patients requiring Ladd procedure declines drastically after the first year of life, and reaches a probability of 1 per 10,000 patients after 20 years of life [19]. Moreover, the gain in quality-adjusted life year (QALY) after a prophylactic Ladd procedure decreases from 0.2 to 0 at the age of 20, and continues negatively as the patient grows older [19]. Therefore, we find a prophylactic procedure unjustifiable, especially if the patient’s age has reached 20 years.

## 4 Conclusion

Intestinal malrotation is an entity that is often missed for years due to its low likelihood in the adult population and nonspecific symptoms. It is often diagnosed with a CT abdomen and is corrected via Ladd procedure, which can be done by laparotomy or laparoscopy. It is crucial for the operating surgeon to identify associated abnormal vascular anatomy and tailor the surgery accordingly.

## Declaration of Competing Interest

All authors declare that there is no financial or personal conflict of interest related to this work.

## Funding

This research did not receive any specific grant from funding agencies in the public, commercial, or not-for-profit sectors.

## Ethical approval

Ethical approval was exempted by our institution.

## Consent

Written informed consent was obtained from the patient for publication of this case report and accompanying images. A copy of the written consent is available for review by the Editor-in-Chief of this journal on request.

## Registration of research studies

NA.

## Guarantor

NA.

## Provenance and peer review

Not commissioned, externally peer-reviewed.

## CRediT authorship contribution statement

**Hassan Dehaini:** Writing - original draft, Writing - review & editing. **Rakan Nasser Eldine:** Writing - original draft. **Samer Doughan:** Editing manuscript. **Mohammad Khalifeh:** Editing manuscript. **Hala Khasawneh:** Writing - original draft. **Hero Hussain:** Editing manuscript. **Eman Sbaity:** Supervision, Writing - review & editing.

## References

[1] Torres A.M., Ziegler M.M. (1993). Malrotation of the intestine. World J. Surg..

[2] Coppola C.P., Coppola C.P., Kennedy J.A.P., Scorpio R.J. (2014). Malrotation. Pediatr. Surg. Diagnosis Treat..

[3] Berseth C.L., Taeusch H.W., Ballard R.A. (1998). Disorders of the intestines and pancreas. Avery’s Dis. Newborn.

[4] Nehra D., Goldstein A.M. (2011). Intestinal malrotation: varied clinical presentation from infancy through adulthood. Surgery.

[5] Agha R.A., Borrelli M.R., Farwana R., Koshy K., Fowler A.J., Orgill D.P., Zhu H., Alsawadi A., Noureldin A., Rao A., Enam A., Thoma A., Bashashati M., Vasudevan B., Beamish A., Challacombe B., De Wilde R.L., Machado-Aranda D., Laskin D., Muzumdar D., D’cruz A., Manning T., Healy D., Pagano D., Goel P., Ranganathan P., Pai P.S., Raja S., Ather M.H., kadioäžlu H., Nixon I., Mukherjee I., Gómez Rivas J., Raveendran K., Derbyshire L., Valmasoni M., Chalkoo M., Raison N., Muensterer O., Bradley P., Roberto C., Afifi R., Rosin D., Klappenbach R., Wynn R., Giordano S., Basu S., Surani S., Suman P., Thorat M., Kasi V. (2018). The SCARE 2018 statement: updating consensus surgical CAse REport (SCARE) guidelines. Int. J. Surg..

[6] Danowitz M., Soulonias N. (2016). Embryology, comparative anatomy, and congenital malformations of the gastrointestinal tract. Edorium J. Anat. Embryol..

[7] Dassinger M.S., Smith S.D., Holcomb G., Patrick Murphy J., Ostlie D. (2014). Malrotation. Ashcraft’s Pediatr. Surg..

[8] Ballantyne G.H., Brandner M.D., Beart R.W., Ilstrup D.M. (1985). Volvulus of the colon. Incidence and mortality. Ann. Surg..

[9] Strouse P.J. (2008). Malrotation. Semin. Roentgenol..

[10] Tackett J.J., Muise E.D., Cowles R.A. (2014). Malrotation: current strategies navigating the radiologic diagnosis of a surgical emergency. World J. Radiol..

[11] Yousefzadeh D.K. (2009). The position of the duodenojejunal junction: the wrong horse to bet on in diagnosing or excluding malrotation. Pediatr. Radiol..

[12] Sizemore A.W., Rabbani K.Z., Ladd A., Applegate K.E. (2008). Diagnostic performance of the upper gastrointestinal series in the evaluation of children with clinically suspected malrotation. Pediatr. Radiol..

[13] Durkin E.T., Lund D.P., Shaaban A.F., Schurr M.J., Weber S.M. (2008). Age-related differences in diagnosis and morbidity of intestinal malrotation. J. Am. Coll. Surg..

[14] Eccleston J.L., Su H., Ling A., Heller T., Koh C. (2016). Gastrointestinal: adult presentation of intestinal malrotation. J. Gastroenterol. Hepatol..

[15] Hagendoorn J., Vieira-Travassos D., van der Zee D. (2011). Laparoscopic treatment of intestinal malrotation in neonates and infants: retrospective study. Surg. Endosc..

[16] Stanfill A.B., Pearl R.H., Kalvakuri K., Wallace L.J., Vegunta R.K. (2010). Laparoscopic Ladd’s procedure: treatment of choice for midgut malrotation in infants and children. J. Laparoendosc. Adv. Surg. Tech. A.

[17] Frasier L.L., Leverson G., Gosain A., Greenberg J. (2015). Laparoscopic versus open Ladd’s procedure for intestinal malrotation in adults. Surg. Endosc..

[18] Graziano K., Islam S., Dasgupta R., Lopez M.E., Austin M., Chen L.E., Goldin A., Downard C.D., Renaud E., Abdullah F. (2015). Asymptomatic malrotation: diagnosis and surgical management: an American Pediatric Surgical Association outcomes and evidence based practice committee systematic review. J. Pediatr. Surg..

[19] Malek M.M., Burd R.S. (2006). The optimal management of malrotation diagnosed after infancy: a decision analysis. Am. J. Surg..

